# Physical and Oxidative Stability of Low-Fat Fish Oil-in-Water Emulsions Stabilized with Black Soldier Fly (*Hermetia illucens*) Larvae Protein Concentrate

**DOI:** 10.3390/foods10122977

**Published:** 2021-12-03

**Authors:** Lucas Sales Queiroz, Federico Casanova, Aberham Hailu Feyissa, Flemming Jessen, Fatemeh Ajalloueian, Italo Tuler Perrone, Antonio Fernandes de Carvalho, Mohammad Amin Mohammadifar, Charlotte Jacobsen, Betül Yesiltas

**Affiliations:** 1 National Food Institute, Technical University of Denmark, 2800 Kongens Lyngby, Denmark; lusaqu@food.dtu.dk (L.S.Q.); feca@food.dtu.dk (F.C.); abhfe@food.dtu.dk (A.H.F.); fjes@food.dtu.dk (F.J.); moamo@food.dtu.dk (M.A.M.); chja@food.dtu.dk (C.J.); 2 Departamento de Tecnologia de Alimentos, Universidade Federal de Viçosa (UFV), Viçosa 36570-900, Brazil; 3 Center for Intelligent Drug Delivery and Sensing Using Microcontainers and Nanomechanics (IDUN), Department of Health Technology, Technical University of Denmark, 2800 Kongens Lyngby, Denmark; faaj@dtu.dk; 4 Departamento de Ciências Farmacêuticas, Universidade Federal de Juiz de Fora (UFJF), Rua José Lourenço Kelmer, São Pedro, Juiz de Fora 36036-900, Brazil; italotulerperrone@gmail.com

**Keywords:** black soldier fly larvae, ohmic heating, ultrasound, emulsifying property, oxidative stability

## Abstract

The physical and oxidative stability of fish oil-in-water (O/W) emulsions were investigated using black soldier fly larvae (BSFL) (*Hermetia illucens*) protein concentrate as an emulsifier. To improve the protein extraction and the techno-functionality, defatted BSFL powder was treated with ohmic heating (BSFL-OH) and a combination of ohmic heating and ultrasound (BSFL-UOH). Fourier transform infrared spectroscopy (FTIR) and differential scanning calorimetry (DSC) were performed in order to characterize the secondary structure and thermal stability of all protein concentrate samples. The interfacial properties were evaluated by the pendant drop technique. The lowest interfacial tension (12.95 mN/m) after 30 min was observed for BSFL-OH. Dynamic light scattering, ζ-potential and turbiscan stability index (TSI) were used to evaluate the physical stability of emulsions. BSFL-OH showed the smallest droplet size (0.68 μm) and the best emulsion stability (TSI = 8.89). The formation of primary and secondary volatile oxidation products and consumption of tocopherols were evaluated for all emulsions, revealing that OH and ultrasound treatment did not improve oxidative stability compared to the emulsion with untreated BSFL. The results revealed the promising application of BSFL proteins as emulsifiers and the ability of ohmic heating to improve the emulsifying properties of BSFL proteins.

## 1. Introduction

Long-chain (LC) fatty acids such as docosahexaenoic acid (DHA), 22:6 (n-3) and eicosapentaenoic acid (EPA), 20:5 (n-3) can be found in fatty fish such as mackerel, tuna, cod fish and salmon, and they are collectively referred to as marine omega-3 (n-3) fatty acids [1]. Notwithstanding, n-3 PUFA-enriched foods have shown important benefits for human health, and they come with the challenge of negatively affecting oxidative stability, since n-3 PUFAs are very susceptible to oxidation.

Fish oil-in-water (O/W) emulsions have been used as an advantageous system for LC n-3 PUFA delivery in order to reduce lipid oxidation [2]. Although emulsions might protect n-3 PUFAs against oxidation, they are thermodynamically unstable [2]. The selection of an appropriate emulsifier plays an important role not only for the physical stability of the emulsion, but also for the oxidative stability. Oxidation in emulsions is initiated at the oil–water interface and factors such as the thickness and charge of the interface can affect the oxidative stability of the emulsion [3]. Therefore, comprehending the structure, properties and inner dynamics of the emulsion interface is essential to improve physico-chemical stability and potentially reduce oxidative effects on food emulsions.

Proteins have been found to protect the oil phase against oxidation due to their chelating and free radical scavenging properties and the ability to form a physical barrier with thick layers at the emulsion interface [4]. The industry has used many synthetic emulsifiers over the last years. Nevertheless, there is a growing demand for natural and more sustainable ingredients in food production due to public health concerns and climate change effects. This demand influences the increasing trend of replacing synthetic and non-sustainable sources of emulsifiers with natural and more eco-friendly protein sources [5].

Among the alternative food sources, edible insects have drawn the attention of the FAO and the scientific community, as it has been reported as a promising protein source with potential technological applications in food systems and a low environmental footprint [5].

*Hermetia illucens*, popularly known as black soldier fly (BSF), has been identified as one of the most promising insects for the commercial production of proteins, showing great benefits for humans and the environment [6]. The protein content of BSFL is stated to be between 37% to 63% of dry matter and is mainly composed of structural and muscular protein [6]. Edible insects can have an increased acceptance by consumers when they are not directly visible in food [7,8].Therefore, the production of insect powder and the exploration of their protein techno-functionalities in food systems have been highlighted as great alternatives to increase consumer’s acceptance [9].

In order to improve these techno-functionalities by altering protein structure, several treatments can be applied, such as ohmic heating [10], microwave [11], ultrasound [12], pulsed electric field [13] and the combination of two or more systems, such as ultrasound and ohmic heating [14].

Ohmic heating (OH) has received increased attention due to its rapid and volumetric heating rates that enable high temperatures to be applied in the sample during a very short time period [15]. During OH treatment, an alternating electric current passes through the sample and its resistance generates an internal heat. A study on the effect of electric field on proteins has stated interesting conformational changes in the proteins’ structure, as it resulted in protein films and gels with distinctive properties [16]. However, there is no particular study reporting the effect of OH on BSFL proteins.

The effect of ultrasound, an acoustic wave method with a frequency of 20–100 kHz, on food structure is explained by the quick formation and collapse of gas bubbles, called ultrasonic cavitation, which is generated through localized pressure differentials that occur in a few microseconds [17]. Ultrasound treatment is reported to affect the structural, physical and functional properties of proteins [18]. In addition, ultrasound has also been applied to insect species such as *Tenebrio molitor*, *Gryllus bimaculatus* and *Bombyx morri* during the protein extraction step, and the study showed that protein yield increased for all insect samples after ultrasound treatment [19].

The objective of this study was to investigate the effect of OH treatment and the combination of OH and ultrasound on BSFL protein extraction and how it would affect the physical and oxidative stability of 5% fish oil-in-water emulsions prepared with BSFL protein concentrate as an emulsifier. Sodium caseinate (CAS) was used as a reference emulsifier in all tests. OH and ultrasound techniques were applied as an assisted extraction method based on the hypothesis that these techniques could increase the yield of protein during the extraction. In addition, both treatments are expected to alter the tertiary and secondary structure of proteins, which can influence the interfacial properties.

## 2. Materials and Methods

### 2.1. Material

Cod liver oil was provided by Vesteraalens A/S (Sortland, Norway) and kept at −40 °C until used. Cod liver oil showed peroxide value of 0.25 ± 0.07 meq peroxides/kg oil. The fatty acid composition (%, *w*/*w*) was found as: C14:0 (4.0), C16:0 (9.2), C16:1n-7 (8.3), C18:0 (2.2), C18:1n-9 (15.8), C18:1n-7 (4.1), C18:2n-6 (2.5), C18:3n-3(0.2), C20:1n-9 (11.4), C20:5n-3 (8.8), C22:1n-11 (5.4) and C22:6n-3(11.4). Alpha-, beta-, gamma-, and delta-tocopherol contents were 146 ± 7, 0 ± 0, 97 ± 2, 43 ± 0.3 μg toc/g oil, respectively. Sodium caseinate (Miprodan 30) with 93.5% protein content was provided by Arla Foods Ingredients amba (Viby J, Denmark).

The BSFL flour was kindly provided by nextProtein, Grombalia, Tunisia. BSFL were fed on formulas of different mixtures based on biowaste according to EU regulations. When harvesting stage was reached, the larvae were separated from the subtrate by sieving. Samples were blanched in hot water at 70 °C for 5 min and softly dried for 3–4 h to reach a moisture content of <7%. The oil fraction was then extracted by pressing the dried sample. A protein meal, partially defatted, was then obtained. Protein-enriched sample was then ground in order to produce the insect flour. The chemicals were purchased from Sigma-Aldrich (St. Louis, MO, US).

Black soldier fly larvae protein concentrate (BSFL) has gone through two different pretreatments in this work: BSFL pretreated with ohmic heating (BSFL-OH) and BSFL pretreated with ultrasound and ohmic heating (BSFL-UOH). All process and analysis are explained within the next sections.

### 2.2. Sample Preparation

#### 2.2.1. Preparation of Defatted Insect Powder

In order to remove the remaining lipids from BSFL flour, it was mixed with ethanol (ratio 1:20, *w/v*) and stirred for 12 h at 40 °C. The sample and the liquid fraction (fat extract) were recovered with a filter paper and decanted, and the fat extract (N° 00H, 7 cm, Sweden) solvent was evaporated using a rotary evaporator (Laborota 4000 efficient, Heidolph Instruments GMBH & CO KG), solvent was recovered and reused in the second extraction to remove any residual fat. Residual solvent was removed by placing the sample under a fume hood overnight.

#### 2.2.2. Ohmic Heating Pre-Treatment

As a pre-treatment step, laboratory scale ohmic heater (BCH Ltd., Whitworth, Lancashire, UK) was used on the defatted BSFL sample. The equipment consisted of a control unit with a maximum supply of 230 V at 60 Hz and a rectangular polyethylene heating cell with two titanium electrodes placed at two ends. For this study, the distance between electrodes and inner width of cell were set to 12 cm and 9.5 cm, respectively. The electric current and voltage were registered by a built-in data logger system, and temperature was monitored using a K-type thermocouple connected with TC-08 data logger (Pico Technology, St. Neots, Cambridgeshire, UK).

Right after the defatting step, the BSFL powder was stirred for 1 h in a NaOH (0.25 M) solution, and 250 mL was transferred to OH cell. The applied voltage was set to 115 V, and the sample was heated to a temperature of 90 °C and held for 15 min. Immediately after the treatment, the treated BSFL was cooled down to room temperature, and the alkaline extraction took place (Section 2.2.4).

#### 2.2.3. Ultrasound and Ohmic Heating Pre-Treatment

Defatted sample (Section 2.2.1) was stirred for 30 min in NaOH solution (0.25 M), followed by ultrasound treatment (Branson, Danbury, CT, USA) in a pulse mode (3 s ON, 3 s OFF) at 20 kHz, 440 W for 15 min.

The intensity of the treatment was determined for each sample using Equations (1) and (2) [20]:

(1) $$Pa=M.\;Cp.\;\left(\frac{dT}{dt}\right)$$

(2) $$I\alpha =\frac{Pa}{Sa}$$

where Pa is power (W), M is the mass 10 g (g), Cp is the specific heat 4.18 (J/g °C) and *dT* and dt are the differential of temperature (°C) and time, respectively, during the first 30 s. Iα is the intensity of treatment, which is equal to the power (Pa) divided by the surface area of 0.69 cm^2^ (Sa) [21]. *Iα* The power and intensity of treatment were found to be 13 W and 18.9 W/cm^2^, respectively.

After ultrasound treatment the sample went through ohmic heating treatment as reported in (Section 2.2.2), and finally, the alkaline extraction (Section 2.2.4) took place.

#### 2.2.4. Alkaline Extraction of Protein

Alkaline protein extraction of BSFL occurred according to Queiroz et al. [6] with slight modifications. The extraction was performed for black soldier fly larvae ohmic heating-treated sample (BSFL-OH), black soldier fly larvae ultrasound and ohmic heating (BSFL-UOH) and the untreated black soldier fly larvae (BSFL). Briefly, the defatted powder was solubilized in 0.25 M NaOH (ratio 1:20, *w*/*v*) and stirred (Polymix Buch & Holm, A/SHerlev, Denmark) at 300 rpm, 40 °C for 1 h. After centrifugation (Sigma Laborzentrifugen GmbH, Osterode am Harz, Germany) at 2500× *g* 4 °C for 20 min, a 2nd alkaline extraction was repeated on the remaining pellet. The isoelectric point, 4.3~4.5, was reached with 2M HCl, in order to promote protein decantation. The solution was left overnight for complete precipitation of the proteins. After decantation, sample was centrifuged at 1300× *g* 4 °C for 15 min to recover the pellet. The pellet was washed twice with distilled water and freeze-dried overnight.

### 2.3. Protein Content

The protein content of BSFL, BSFL-OH and BSFL-UOH was measured according to Dumas method. A nitrogen-to-protein conversion factor (Kp) of 5.62 was used according to Janssen et al. [22].

### 2.4. Protein Characterization

#### 2.4.1. Differential Scanning Calorimetry (DSC)

DSC 250 (TA Instruments, New Castle, DE, USA), with a Refrigerated Cooling System 90, was used in order to comprehend sample’s thermal stability. Distilled water (melting point (m.p.) = 0 °C; DHm = 334 J/g) and indium (m.p. = 156.5 °C; DHm = 28.5 J/g) were used for calibration. As reference, empty aluminum pans (20 μL volume), hermetically sealed, were used. Nitrogen was used as the carrier gas, with a flow rate of 50 mL/min. The DSC was used to measure the thermal stability of all protein concentrate samples produced in this study. An amount of 7 mg of sample was weighed in duplicate. First, the system was cooled to 0 °C and then scanned from 0 °C to 250 °C at a heating rate of 5 °C/min, The thermograms (heat flow as function of the temperature) all the characteristic peaks, glass transition, thermal unfolding and solid-melting were identified, according to Al-Saidi et al. [23]. Trios software^®^ (New Castle, DE, USA) was used for data interpretation.

#### 2.4.2. Fourier Transform Infrared Spectroscopy (FTIR)

FTIR spectra of all protein concentrate samples were stated using a PerkinElmer Spectrum 100 spectrometer (Waltham, MA, USA), based on a Universal Attenuated Total Reflectance sensor 125 (UATR-FTIR). The transmission mode was applied in a range of 4000–650 cm^−1^. All the spectra were plotted as absorbance (AU) in function of wavenumber (cm^−1^). All the measurements were performed in triplicate.

### 2.5. Interfacial Tension of BSFL Protein Concentrate at the Oil–Water Interface

The interfacial properties of each emulsifier in oil–water interface were verified by the pendant drop technique, using a drop tensiometer OCA20 (DataPhysics Instruments, Filderstadt, Baden-Württemberg, Germany). For that, 0.01% (*w*/*w*) of each protein concentrate, BSFL, BSFL-OH, BSFL-UOH and sodium caseinate (CAS) was prepared separately in 10 mM sodium acetate-10 mM imidazole buffer (pH 7.0) and was stirred for 2 h. The syringe was filled with the aqueous phase, and for each analysis a small drop was formed using the automated syringe with quartz glass cuvette. The cuvette contained MCT oil (WITARIX^®^ MCT 60/40, IOI Oleo GmbH, Hamburg, Germany). The calculation of the interfacial tension was based on the shape analysis of a pendant drop according to the Young–Laplace equation, Equation (3):

(3) $$\Delta P=\gamma \left(\frac{1}{\;\;R_{1}}+\frac{1}{R_{2}}\right)$$

where ΔP (mN/m^2^) stands for the difference in pressure across the interface, γ (mN/m) is the interfacial tension, and $R_{1}$ and $R_{2}$ (m) are the principal radii of curvature of the pendant drop. All the measurements were performed in duplicate.

### 2.6. Emulsion Preparation and Storage Experiment

Emulsifiers (CAS, BSFL, BSFL-OH, BSFL-UOH) were dissolved in a 10mM sodium acetate-10 mM imidazole buffer and placed in a shaking water bath at 50 °C for 2 h. Then, solutions were left stirring overnight at 300 rpm at room temperature. Before emulsification, pH was adjusted to 7 using 2 M NaOH. Emulsions were produced with 5% oil and 0.2% protein in 220 g batches. To produce the emulsion each solution was pre-homogenized using ultraturrax (Polytron, PT120E, 18,000 rpm, 30 s) for 3 min at 16,000 rpm. All the oil was added during the first minute. Then, homogenization was carried out in a microfluidizer [24], pressure 9 Kpsi and 3 passes. In order to accelerate lipid oxidation and prevent microbial growth, iron solution (Fe^2+^, 50 µM) and sodium azide (0.05% *w*/*v*) were added, respectively. Emulsions were stored in amber bottles at room temperature in darkness. Physical and oxidative stability data were obtained at days 0, 1, 3, 6 and 10.

### 2.7. Physical Characterization of Emulsions

#### 2.7.1. Turbiscan Analysis

Physical stability was evaluated using Turbiscan Tower (Formulaction, Toulouse, France). The technology is based on the static multiple light scattering principle. Samples were illuminated by an infrared light source (λ = 880 nm), and two sensors collected the backscattered (BS) and transmitted (T) signals. The stability criteria were based on the Turbiscan Stability Index (TSI) according to a previous work [20]. The TSI is a number calculated at time *t* by summing up all temporal and spatial variations in a considered zone:

(4) $$TSI_{\left(t\right)}=\frac{1}{N_{h}}\overset{t_{max}}{\underset{t_{i}=1}{\sum }}\;\overset{z_{max}}{\underset{z_{i}=z_{min}}{\sum }}\left|B\right.ST\left(t_{i},z_{i}\right)-BST(t_{i-1},z_{i}\left.)\right|$$

with: $t_{max}$ is the measurement point corresponding to the time *t* at which the TSI is calculated, $z_{min}$ and $z_{max}$ are the lower and upper selected height limits, respectively, $N_{h}$ = (Z_max_ − Z_min_)/Δh is the number of height positions in the selected zone of the scan, and BST is the considered signal (BS if *T* < 0.2%, *T* otherwise). Therefore, the lowest the *TSI* is the most stable sample.

Samples were analyzed for the first 24 h and then from day 3 to day 7 in order to verify the stability over time. All samples were stored at room temperature and protected from light. A total of 67 scans were performed during the first 24 h, and 269 were performed from day 3 to day 7; the number of scans were more frequent during the first hours in order to see changes in more detail.

#### 2.7.2. Hydrodynamic Diameter (Dh) and ζ-Potential

Droplet size of the emulsions was measured using laser diffraction in a Mastersizer 2000 (Malvern Instruments, Ltd., Worcestershire, UK) on days 0 and 10 according to Horn et al. [25]. Results were measured based on the surface area moment mean (D(3,2)), as given in Equation (5):

(5) $$D\left(3,2\right)=\frac{\Sigma n_{i}d_{i}^{3}}{\Sigma n_{i}d_{i}^{2}}$$

where n corresponds to number of droplets with measured diameter, d is the diameter of the droplet, and i represents the size class of the droplets. Samples were measured in duplicate.

The analysis was performed on the emulsions using Zetasizer Nano ZS (Malvern Instruments, Malvern, Worcestershire, UK) with capillary cells. ζ-Potential was calculated from the electrophoretic mobility (μ) using the Henry equation, Equation (6):

(6) $$\zeta \;=\frac{3\eta \mu }{2\epsilon f\left(kRh\right)}\;\;\;$$

where η is the viscosity of the buffer (1.033 × 10^−3^ Pa s^−1^), *ϵ* is the medium dielectric constant (dimensionless), Rh is the complex radius (nm) and f(kRh) is Henry’s function. A value of 1.5 was adopted for f(kRh), referred to as the Smoluchowski approximation, as the analysis was performed in aqueous media.

### 2.8. Oxidative Stability of Emulsions

#### 2.8.1. Primary Oxidation Products—Peroxide Value (PV)

Primary oxidation products were determined on lipids extracted from the emulsion according to the Bligh and Dyer method with slight changes [26]. Lipids were extracted using 10 g of emulsion and 30.0 mL of methanol and chloroform, 1:1. First, the 30 mL of methanol was added, followed by 15 mL of chloroform, and mixed at 8000 rpm for 10 s with an Ultraturrax (Polytron, PT120E). Another 15mL of chloroform was added and mixed again for more 10 s at 8000 rpm. Finally, 15 mL of water was added and mixed for another 10 s at 8000 rpm. Samples were centrifuged at 2800 at 18 °C for 10 min. The water phase was discarded, and chloroform phase was filtered and weighed, and 9 g was deposited into a previously tared beaker. The beaker with chloroform extract was left overnight in the fume hood and then dried in the oven at 105 °C for 2 h and weighed. PV was subsequently determined on the lipid extracts by colorimetric determination of iron thiocyanate on a spectrophotometer (Shimadzu, UV mini 1240, Kyoto, Japan) at 500 nm [3]. In sum, Bligh and Dyer extracts were weighed and left to evaporate to dryness under N_2_ flow. An amount of 10 mL of chloroform/methanol 7:3 (*v/v*) was added to all samples, including 2 blank tubes. Two other tubes with Fe (III) were included as standard samples. An amount of 50 μL of thiocyanate solution was added to all tubes, and then 50 μL of Fe (II) chloride solution was added to all tubes except for the blank ones. All tubes were incubated for 5 min at room temperature in darkness, and finally, the absorbance was measured in the previously mentioned spectrophotometer. Measurements were conducted in duplicate.

#### 2.8.2. Tocopherol Content—HPLC

HPLC (Agilent 1100 Series; Column: Waters Spherisorb 3 μm Silica; 4.6 × 150 mm) was used to analyze tocopherols in all emulsions. Tocopherol analysis was performed according to the AOCS Official Method [27] using lipid extracts (Section 2.8.1), and samples were later evaporated and dissolved in heptane. All the nalyses were performed in duplicate. Tocopherol content was expressed in mg/kg of sample weight. The method analyzes all 4 tocopherol homologues. The quantification is based on the integrated area in the chromatogram.

#### 2.8.3. Secondary Oxidation Products—Dynamic Head Space GC-MS

Volatile compounds were measured according to García-Moreno et al. [28]. Volatile compounds were trapped on Tenax GR tubes by dynamic headspace using the following purging conditions: temperature of 75 °C, 30 min and nitrogen flow 150 mL/min. The volatile compounds were separated in a gas chromatograph (Agilent Technologies, 6890 N Network GC System, DE, USA) on a 30 m DB 1701 fused silica capillary column (0.25 mm i.d., 1 µm film thickness; Agilent Technologies, J&W GC Columns, Palo Alto, CA, USA). In order to identify individual volatile compounds, the MS library searches (Wiley 138 K, John Wiley and Sons, Hewlett-Packard) and mass-spectrometry (Agilent 5973 Network Mass Selective Detector, Agilent Technologies, 70 eV; mass-to-charge ratio scan between 30 and 250) were used. The volatile compounds 2-ethyl-furan,1-penten-3-one, pentanal, (*E*)-2-pentenal, 1-penten-3-ol, hexanal, (*E*)-2-hexenal, *N-heptanal*, (z)-4-heptenal,* (E)*-2-heptenal, 1-octen-3-ol, octanal, *(E,E)*-2,4-heptadienal, 2-octenal and *(E,E)*-2,4-decadienal were determined in emulsion samples. A stock standard solution was prepared with all aforementioned volatile compounds, and seven different concentrations of diluted stock solution were added into fish oil-in-water emulsion. This method was performed in order to maintain similar release conditions for standard volatile compounds. Analysis was performed in triplicate, and the results are stated as ng/g of emulsion.

### 2.9. Statistical Analysis

In the present study, ANOVA, followed by Tukey’s test (*p* < 0.05 as the level of significance) was used for all the statistical analyses. Data were treated by Statistical Package for the Social Sciences software (SPSS 22.0, SPSS Inc., Chicago, IL, USA).

## 3. Results and Discussion

### 3.1. Protein Content of BSFL before and after Pre-Treatments

The conventional alkaline extraction method was able to provide samples with a protein content of 62 ± 0.2%. A similar result, using the same method of extraction, was obtained previously by Queiroz et al. [6]. In order to increase the yield of extraction and to improve the protein functionality, ohmic heating and ultrasound techniques were applied. Using ohmic heating as a pre-treatment on the insect flour (BSFL-OH), the yield of protein extraction reached 66.7 ± 0.1%, and using the combination of ohmic heating and ultrasound (BSFL-UOH), the protein content was equal to 66.2 ± 0.1%. A previous work using ultrasound was able to improve protein extraction from edible insects [19]. Therefore, herein we aimed to implement both techniques to optimize the protein extraction. However, when using ohmic heating or a combination of both ultrasound and ohmic heating, the yield of extraction was similar and not statistically different (*p* < 0.05). This was the first time that ohmic heating was applied to a BSFL sample in order to increase the protein content. Therefore, different parameters might play an important role in increasing the yield of extraction when using ultrasound and ohmic heating.

### 3.2. Differential Scanning Calorimetry (DSC)

**Figure 1:**
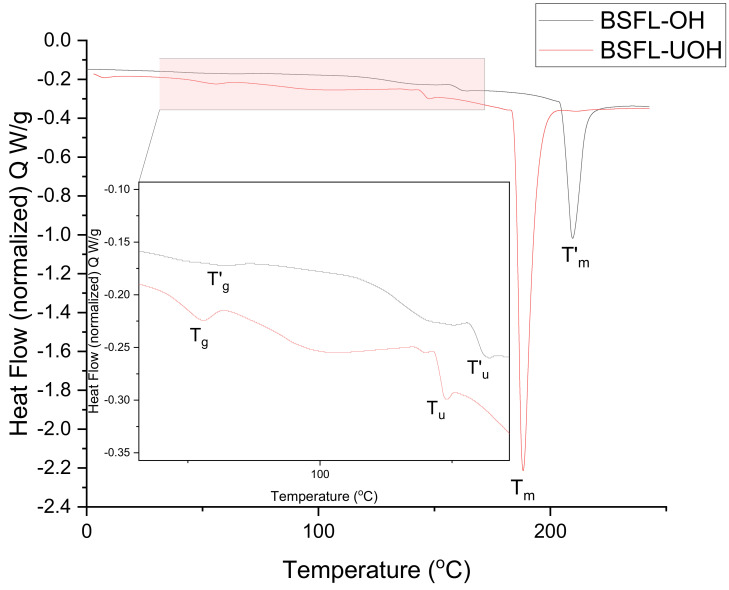
DSC thermograms of BSFL-OH and BSFL-UOH protein concentrate.

Qualitative and quantitative information can be obtained through thermal stability analysis, and DSC has been largely used for the characterization and identification of phase transitions in food products [29]. Normally, phase transition is grouped in two types: first-order transition, which includes crystallization, protein denaturation, melting, condensation and evaporation, and second-order transition, commonly represented by the glass transition temperature [30]. Thermograms are reported as heat flow (W/g) in the function of temperature (°C). The DSC graph for the BSFL-OH and BSFL-UOH samples is reported in Figure 1. The thermogram was marked with T_g_, T_u_, T_m_ (BSFL-UOH) and T’_g_, T’_u_, T’_m_ (BSFL-OH) for the glass transition, unfolding and solid melting point, respectively. The glass transition was observed as a shift in the curve between 45–60 °C for both BSFL-UOH (T_g_ = 48 °C) and BSFL-OH (T’_g_ = 56 °C). The results are similar to those of previous studies reporting on *H. illucens* protein extract [6]. An endothermic peak, identified as an unfolding peak, T’_u_ and T_u_, can be observed at 162 °C and 143 °C for BSFL-OH and BSFL-UOH, respectively. The same region has been stated as the thermal unfolding point for other proteins [23], including black soldier fly larvae, which has shown to have an unfolding point at 150 °C for BSFL protein extract [6]. Unfolding involves the dissociation of intramolecular bonds, which results in an endothermic process. On the other hand, protein aggregation involves the possible reformation of intra- and intermolecular hydrophobic bonds resulting from different treatments, such as sonication [31]. In this study, there was a decrease in the denaturation temperature for the sample treated with OH, after which sonication occurred. In addition, an increase in enthalpy was observed for the BSF-UOH sample, probably due to protein aggregation and bond formation. These data are consistent with a previous study on whey protein concentrate that reported protein aggregation and increase in enthalpy after prolonged sonication treatment [32]. The same pattern can be observed in the melting point (T_m_), as an increase in enthalpy and decrease in temperature was observed for BSFL-UOH, which might be due to the long exposure time of the samples to ultrasound treatment, causing protein aggregation as similarly reported in a previous study for pea protein and fish skin gelatin [20].

### 3.3. Fourier Transform Infrared Spectroscopy (FTIR)

**Figure 2:**
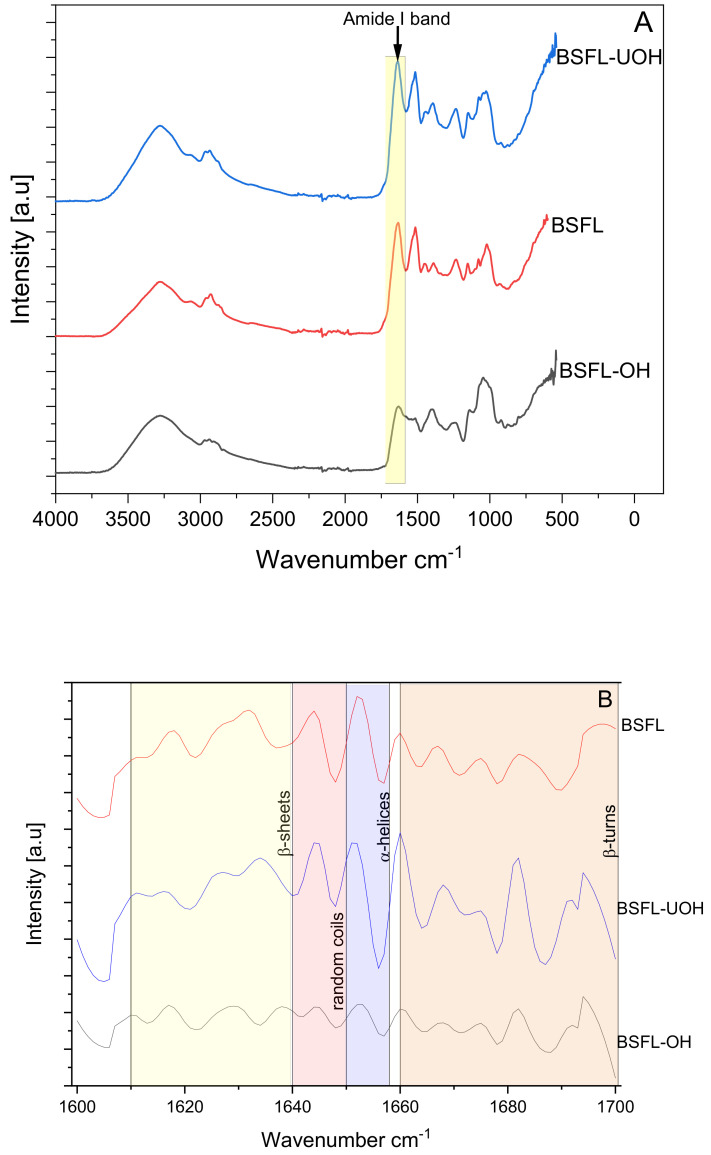
(**A**) FTIR spectra of BSFL, BSFL-OH and BSFL-UOH and (**B**) second derivative of amide I band, different colors yellow, red, purple, and orange represent the different secondary structure ranges β-sheets, random coils, α-helices and β-turns, respectively.

In order to characterize the protein concentrate samples and verify how the OH and ultrasound pre-treatment can alter the tertiary and secondary structure of BSFL proteins, the samples were analyzed by FTIR (Figure 2A). A previous work characterized the BSFL protein concentrate, obtained by the same alkaline extraction as herein reported, and a high absorbance was noticed at 1634 cm^−1^, which was assigned to the β-sheet structure [6]. Another work reported the main amide bands and specifically the amide I, which is correlated to the secondary structure of proteins, highlighting the presence of α-helices (1650–1658 cm^−1^), β-sheets (1610–1640 cm^−1^), random coils (1640–1650 cm^−1^) and β-turns (1660–1695 cm^−1^) according to the peaks on each wavenumber range [33]. Similarly, the present work identified the amide bands, and amide I was reported to show some differences after the pre-treatments. BSFL and BSFL-UOH showed a more similar FTIR spectrum in the range of 1600–1700 cm^−1^. However, BSFL-OH, instead, had a lower and diffuse peak distribution, which highlights changes in the secondary structure of the proteins after OH treatment only. The peaks at 1647 cm^−1^ (amide I) and 1538 cm^−1^ (amide II) are indicative of α-helices conformation, and the presence of shoulder bands at 1626 cm^−1^ and 1517 cm^−1^ also indicates some degree of β-sheet secondary structure [34]. A more detailed analysis can be seen in Figure 2B, where the Savitsky–Golay method and the second derivative were applied, similarly to a previous study on the protein concentrate of the black soldier fly [35]. The spectrum shows a qualitative analysis of the amide I region, correlated to C=O stretching. It was possible to state the different distribution of each secondary structure on the proteins extract where BSFL-OH showed a very clear different pattern of all secondary structure when compared to BSFL and BSFL-UOH. The peaks in the range of β-sheets are similar between BSFL-UOH and BSFL but showed a different pattern when both were compared to BSFL-OH. In addition, the intensity of peaks for α-helices and random coils appeared to be lower for BSFL-OH. The β-turns region was observed to be more affected by ultrasound treatment, whereas BSFL-UOH had a sharper peak with a different distribution when compared to BSFL and BSFL-OH. The secondary structure of proteins is directly connected to its functionalities, including its solubility and emulsifying properties [36].

Amide II band at 1520 cm^−1^, which derives from in-plane N-H bending and CN stretching vibration, could be noticed more clearly for BSFL and BSFL-UOH than for BSFL-OH. Amide III band was identified at 1234 cm^−1^ for BSFL and BSFL-UOH, but a slight shift was observed for BSFL-OH at 1250 cm^−1^. This region is related to the combination of C-N stretching vibrations and N-H deformation from amide linkages, absorptions from CH_2_ wagging vibrations, normally arising from glycine and proline side-chains [6]. Due to the complexity of each extract, other bands can be assigned to the presence of C-O bonds from carbohydrates (900–1200 cm^−1^) and the presence of a-chitin by the bands between 1500 and 1700 cm^−1^ [37,38].

### 3.4. Emulsion Characterization

#### 3.4.1. Interfacial Tension

**Figure 3:**
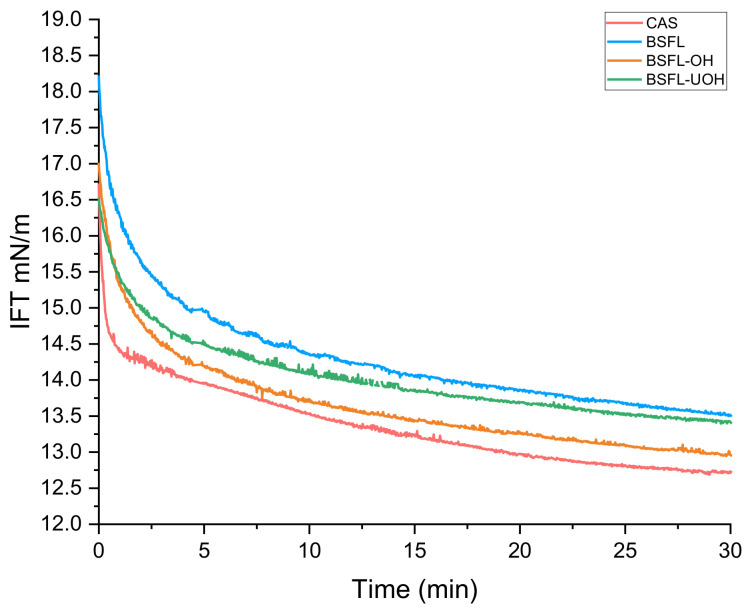
Interfacial tension of CAS (sodium caseinate), BSFL (Black soldier fly larvae), BSFL-OH (black soldier fly larvae after ohmic heating) and BSFL-UOH (black soldier fly larvae after ohmic heating and ultrasound) emulsifier samples. The oil–water interfacial tension without emulsifier was 25 mN/m during 30 min for MCT oil/water.

Oil–water interfacial tension (IFT) was measured in order to understand the effects of different methods of protein extraction compared to CAS and conventional alkaline extraction. Figure 3 represents interfacial tension as a function of time for the different samples BSFL, BSFL-OH, BSFL-UOH and CAS. During the first 3 min, the IFT quickly dropped for BSFL-OH, from 17.0 to 14.4 mN.m^−1^, which means the proteins had a fast adsorption to the oil–water interface, probably due to the smaller peptide size and the pattern of distribution of hydrophilic and hydrophobic groups on the protein surface. The BSFL-UOH and BSFL samples showed a slower reduction in interfacial tension and reached a higher IFT after 30 min, compared to BSFL-OH (12.9 mN.m^−1^) and CAS (12.7 mN.m^−1^). The results corroborate the data obtained from the Turbiscan emulsion stability analysis, where BSFL-OH showed the most promising result. The data were also comparable to CAS, which was the reference emulsifier. Besides protein size and the disposition of hydrophobic groups on the protein surface, lipid oxidation can also induce changes in the interfacial composition, as surface-active molecules are formed in the oil phase [39]. A more uniform heat provided by OH without inducing excessive denaturation or coagulation of proteins might have influenced the best performance of BSFL-OH [40]. The low interfacial tension found for BSFL-OH implies a superior interface activity and a minimized contact area between hydrophobic and hydrophilic regions. In general, the dynamics of the oil–water interfacial tension is managed by the diffusion rate of the emulsifier and the time it takes to reach and adsorb to the interface. In addition, the clearer differences in secondary structure for BSFL-OH, reported by FTIR analysis, might help us to understand the more promising results compared to BSFL and BSFL-UOH, as the disposition of different secondary structures can directly affect protein functionality.

A previous work reported the interfacial properties of black soldier fly protein concentrate (BSFPC) using a sunflower oil–water system to measure the decrease in IFT. The study reported that BSFCP instantaneously decreased IFT to 8.4 mN/m and reached 3.4 mN/m after 90 min of experiment [35]. The author used a 10 times higher protein concentration, 0.1% (*w*/*w*), compared to the concentration used in this study, 0.01% (*w*/*w*). The higher protein concentration, time of analysis and the difference between the method of extraction in both works are some of the features that might have affected the dynamic of the interfacial properties of the studied proteins.

#### 3.4.2. Turbiscan Emulsion Stability Analysis

**Figure 4:**
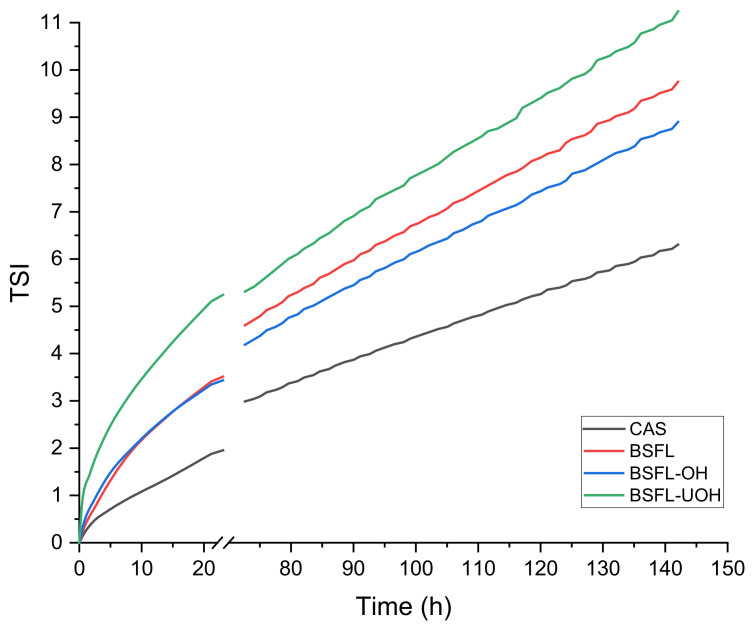
TSI analysis of CAS (sodium caseinate), BSFL (black soldier fly larvae), BSFL-OH (black soldier fly larvae after ohmic heating) and BSFL-UOH (black soldier fly larvae after ohmic heating and ultrasound) emulsions as a function of time (hours).

In order to quantitatively compare the emulsion stability for all samples, the TSI as a function of time was applied (Figure 4). CAS emulsion was used as a reference and showed the highest stability during the whole experiment, followed by BSFL-OH, BSFL and BSFL-UOH emulsions. During the first 17 h, BSFL emulsion showed comparable stability with BSFL-OH emulsion; however, after this point, BSFL-OH emulsion showed improved stability but with no statistical difference (*p* < 0.05) compared to BSFL emulsion. BSFL-UOH emulsion showed the lowest stability over time, which was statically significant (*p* < 0.05) when compared to BSFL, BSFL-OH and CAS emulsions, possibly due to the exposure of hydrophobic groups and increase in surface hydrophobicity after the ultrasound treatment, which could cause an increase in protein aggregation, as previously stated by some studies [12,41]. This fact might have hampered the stabilization of the droplets in emulsion. BSFL-UOH emulsion reached a TSI equal to 3 after the first 8 h, while BSFL-OH emulsion and BSFL emulsion reached this value after 18 h and 17 h, respectively. A TSI lower than 3 is considered as a reference value for a stable system [20]. CAS emulsion reached the value of 3 only after 74 h. In conclusion, the more uniform heating and higher protein content obtained from OH treatment was good for providing better emulsifying stability that was statistically significant (*p* < 0.05) for O/W emulsions compared to sample treated by the combination of the ultrasound and OH and a slight improvement, which was not statistically significant (*p* < 0.05), when compared to the untreated sample at the end of the experiment. A previous study on soybean milk protein reported an increase of 38% in emulsifying activity after the sample was treated with OH-assisted extraction [42]. A study comparing black soldier fly protein concentrate (BSFPC) with whey protein isolate (WPI) showed that at a low concentration (0.1%), BSFPC has stronger emulsifying properties than WPI, and at higher protein concentration (2%), BSFPC revealed comparable emulsion activity to WPI. In addition, it has been reported that sunflower oil-in-water emulsion, stabilized by BSFPC, had a similar result to emulsion stabilized with WPI. Yet, BSFPC successfully reduced the mean droplet diameters of emulsions prepared with 20% to 40% of lemon oil compared to the same emulsions prepared with WPI [35].

The ability of a protein to stabilize emulsions could be an important characteristic required in many food formulations. Therefore, different strategies can be considered in order to improve this. A study reported on the emulsifying properties of BSFL protein sample and its hydrolyzed products by different enzymes. The study revealed that the non-hydrolyzed sample showed improved emulsifying stability compared to the sample hydrolyzed by alcalase, (DH = 18.4%), papain (DH = 15.34%) and pepsin (DH = 9.8) [43].

#### 3.4.3. ζ-Potential and Hydrodynamic Diameter

**Figure 5:**
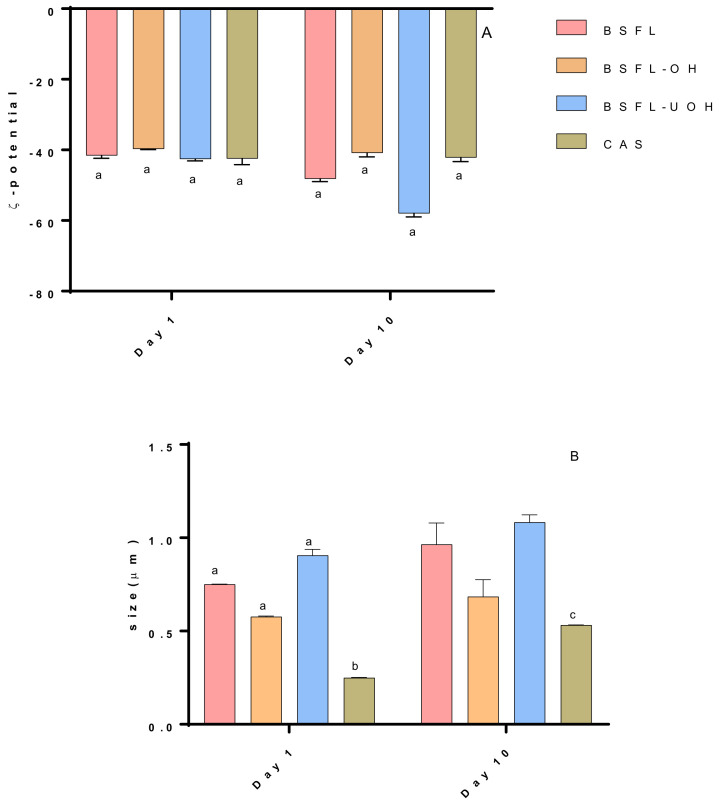
(**A**) ζ-potential (mV) measured during 10 days of storage and (**B**) size (μm) measured during 10 days of storage. Different letters mean a significant difference for *p* < 0.05 among samples.

The emulsion stability relies on a variety of factors, including particle charge and hydrodynamic diameter, which can play an important role. ζ-potential is defined as the potential difference between the surrounding medium and the stationary layer of fluid attached to the particle [44]. A numerically ζ-potential values close to ±30.00 mV is considered an indication of stability, as particles will tend to repel each other and might avoid coalescence or general aggregation [45,46]. ζ-potential was calculated on day 1 and day 10 after emulsions were produced (Figure 5A). After the first day, all BSFL emulsions showed a high zeta potential, between −39 and −42 mV, which can be interpreted as sufficient electrostatic repulsion to prevent droplet coalescence. The values were similar to the reference CAS emulsion, −42 mV. A similar zeta potential value has previously been reported (−31 mV) for oil-in-water emulsion stabilized with CAS [47]. Wang et al. [35] studied emulsions stabilized with BSFL protein concentrate and reported that it had a strong surface repulsive effect, even after seven days of storage, with zeta potentials values between −40 mV to −35 mV. Similarly, no emulsions showed significant changes in zeta potential over 10 days of storage, except for BSFL-UOH emulsion, which increased the zeta potential to −58 mV. This increase in surface charge might be caused by the oxidation of the oil phase, which will release chemical compounds to the interface and can promote chemical changes and physical destabilization [20,48].

The droplet size results are reported in Figure 5B. Globally, droplet surface size has not exceeded 0.90 μm in the first day of storage. CAS emulsion showed the lowest droplet size (0.24 μm). After ten days of storage BSFL-OH emulsion showed a not statistically significant increase in particle size, from 0.57 μm to 0.68 μm, whereas BSFL, BSFL-UOH and CAS emulsion showed a larger increase reaching 0.96 μm, 1.08 μm and 0.53 μm, respectively. The droplet size herein reported corroborates with the stability data stated in the Turbiscan analysis (Section 3.4.2), where the CAS emulsion with the smallest particle size showed the best stability followed by BSFL-OH, BSFL and BSFL-UOH emulsions after 10 days of storage. The combination of the ultrasound and OH in BSFL-UOH emulsion provided the biggest droplet size compared to the alkaline extraction and OH treatment alone. A previous study by Jiang et al. [49] reported that high-power (450 W) ultrasound treatment on black bean protein isolate led to an increase in particle size due to the repolymerization of aggregates through noncovalent and covalent interactions. Similarly, other studies on egg protein and bovine serum albumin reported that high-intensity ultrasound treatment increased particle size through protein aggregation that was caused by hydrophobic interactions [12,41]. The presence of more protein aggregates can hamper the capacity of proteins to stabilize the O–W interface and then cause the decrease in emulsifying stability.

### 3.5. Oxidative Stability of Emulsions

#### 3.5.1. Primary Oxidation Products

**Figure 6:**
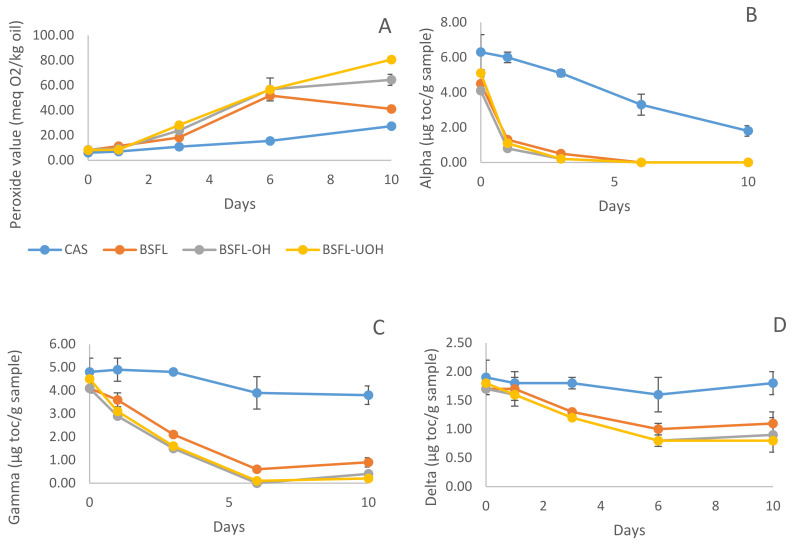
(**A**) Formation of primary oxidation products and consumption of (**B**) alpha-tocopherol, (**C**) gamma-tocopherol, and (**D**) delta-tocopherol content during 10 days of storage.

The oxidation rate of each emulsion was compared during 10 days of storage based on peroxide value (PV) analysis, indicating the formation of lipid hydroperoxides (Figure 6A). After 24 h, CAS, BSF-OH and BSF-UOH emulsions showed similar results for oxidation, 7.12 ± 0.18, 9.36 ± 0.85, 8.50 ± 0.30 meqO_2_/Kg oil, respectively, and BSFL emulsion had a higher value (11.35 ± 0.19 meqO_2_/Kg oil) compared to the others. Until day 6, except CAS, all emulsions had a fast increase in PV and, in general, they had higher PVs compared to CAS emulsion. At day 10 of storage, BSFL-UOH (80.6 ± 0.87 meqO_2_/Kg oil) had a significantly (*p* < 0.05) higher prooxidant effect when compared to BSFL-OH (64.4 ± 4.41 meqO_2_/Kg oil) and BSFL (41.0 ± 1.11 meqO_2_/Kg oil), and all the three emulsions had significantly (*p* < 0.05) higher PV compared to CAS (27.33 ± 0.32) at day 10. One possible explanation might be the low capacity of BSFL-UOH samples to prevent the metals in the aqueous phase from promoting lipid hydroxyl radicals and cause further oxidation of other lipids [50]. In addition, some studies reported that the electrical charge of the oil droplets can impact lipid oxidation, whereas negatively charged particles showed higher lipid oxidation compared to the positively charged ones. It was argued that the positive particles can repel the positive prooxidant ions, while negative particles will attract the same ions [50,51]. This might have contributed to the high oxidation in all samples. In addition, BSFL-UOH showed the largest interface area, which could lead to more oxidation. PV for BSFL (6.2 ± 1.0 meqO_2_/Kg oil), BSFL-UOH (5.4 ± 1.1 meqO_2_/Kg oil) and BSFL-OH (2.8 ± 0.2 meqO_2_/Kg oil) protein concentrate was also evaluated and showed that ultrasound treatment might have led to a higher oxidation compared to OH only.

Conversely, a previous study reported the in vitro antioxidant activity of BSFL protein and protein hydrolysates using different models, including the radical scavenging model (DPPH and ABTS). The results showed that BSFL samples showed promising antioxidant properties, mainly the hydrolyzed ones [52]. However, different methods of protein extraction and treatments were used, which can cause protein structural changes that compromise the antioxidant activity. After 10 days of storage, the BSFL sample showed a decrease in PV from 51.08 ± 2.56 to 41.09 ± 1.11. This can be explained by the decomposition of hydroperoxides by the hemolytic scission of the double bond adjacent to the hydroxyl group to form the secondary oxidation products [53,54].

#### 3.5.2. Changes in Tocopherol Content—HPLC

At day 0, tocopherol homologues levels in the emulsions showed the following range: alpha-tocopherol from 4.10 ± 0.1 to 6.30 ± 1.0, gamma-tocopherol from 4.10 ± 0.1 to 4.80 ± 0.6, and delta-tocopherol 1.70 ± 0.1–1.90 ± 0.3 mg toc/g of sample (Figure 6B–D). As the fish oil content was the same in all samples, the significant reduction in tocopherol contents in samples with BSFL proteins at day 0 was attributed to the consumption of tocopherol during emulsion production. Tocopherol consumption showed a similar behavior for all BSFL emulsions, and CAS emulsion had the highest tocopherol content after 10 days of storage. Alpha-tocopherol significantly decreased after 10 days of storage for all emulsions, with a sharp reduction even during the first day of storage. The results indicate that alpha-tocopherol was acting as an antioxidant in the presence of the BSFL samples and was the most consumed tocopherol when compared to gamma- and delta-tocopherol. Considering the CAS emulsion, the consumption of alpha-tocopherol significantly decreased during 10 days of storage, whereas gamma- and delta-tocopherol content had no significant difference (*p* < 0.05). After 6 days of storage, alpha-tocopherol decreased to 0 for BSFL, BSFL-OH and BSFL-UOH emulsions, and gamma-tocopherol reached 0 for BSFL-OH emulsion. These results are consistent with the increase in peroxide value previously stated for all BSFL protein containing emulsions after 6 days of storage. Therefore, the formation of primary oxidation products during the storage for these emulsions might have been higher if no tocopherols were present and consumed as antioxidants. BSFL emulsion had a slightly higher value for gamma- and delta-tocopherol, which also corroborates with PV value, whereas BSFL emulsion had the lowest oxidation among the three emulsions stabilized by insect protein.

#### 3.5.3. Secondary Volatile Oxidation Products—DHS GC-MS

**Figure 7:**
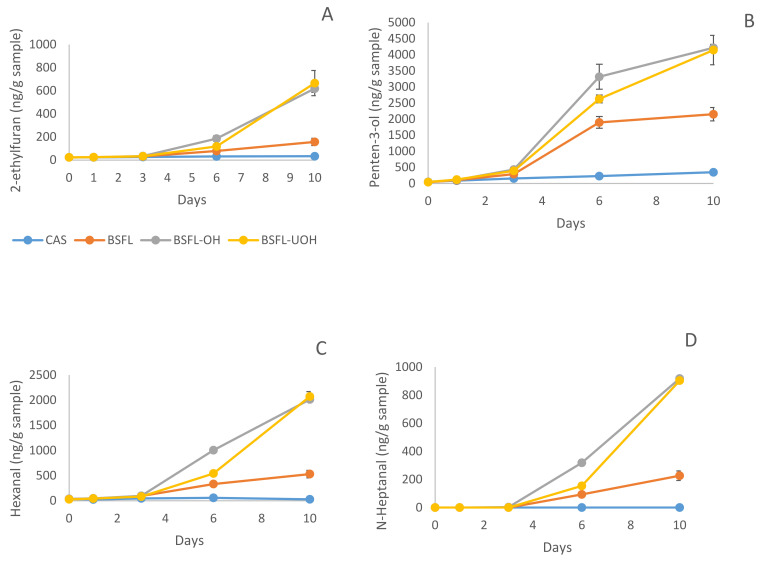
Content of secondary volatile in all emulsion during 10 days of storage: (**A**) 2-ethylfuran content; (**B**) Penten-3-ol content; (**C**) Hexanal content; (**D**) N-Heptanal content.

The formation of volatile compounds was measured during 10 days of storage for all emulsions. Figure 7 shows the concentration of some secondary volatiles present in the emulsions. 2-ethylfuran, penten-3-ol, hexanal and heptanal were chosen due to their higher concentration in the emulsions. 2-ethylfuran and penten-3-ol originated from the oxidation of n-3 PUFAs such as EPA and DHA, while hexanal and heptanal come from the oxidation of omega-6 and omega-9 fatty acids, respectively [55]. The secondary volatile compounds showed a low concentration until the third day of storage for all emulsions with insect protein as emulsifier. After day 3 of storage, a clear increase in the volatile content can be noticed with some differences between emulsions prepared with BSFL, BSFL-OH and BSFL-UOH. CAS emulsion had the lowest volatile content for all compounds analyzed and, for heptanal, CAS emulsion had the concentration, which was equal to 0 during the 10 days of storage. CAS is known for providing a good physical barrier at the oil–water interface and also for its chelating activity, both in the continuous phase and at the o–w interface [56]. This good and homogeneous physical barrier at the interface can efficiently hamper the diffusion of prooxidant molecules when compared to all emulsions containing BSFL proteins.

After 10 days of storage, BSFL emulsion had significantly lower volatiles content (*p* < 0.05) for 2-ethylfuran, penten-3-ol, hexanal and heptanal (including other volatiles shown in the supplementary material, Figure S1) compared to BSFL-OH and BSFL-UOH. The data corroborates the peroxide value previously reported and tocopherol consumption. The result suggests that OH and ultrasound treatments had an important effect in the BSFL proteins extract, since treated samples showed higher oxidation compared to untreated one. A previous study evaluating the effect of OH and ultrasound on whey protein concentrate stated that the application of both pretreatments may alter the protein structure and disrupt some covalent bonds [14]. The unfolding process of proteins and the exposure of hydrophobic groups might cause an increase in hydrophobic interaction between proteins and the formation of aggregates, which can also shield some of the sites with antioxidant activity [57]. The disposition of these aggregates on the oil–water interface can directly influence lipid oxidation since a nonhomogeneous distribution of proteins on the interface can leave spots, whereas prooxidants can enter and oxidase the lipids [57].

The exposure of some nucleophilic amino acids, such as histidine and lysine, can favor covalent reactions between secondary oxidation products and the protein, which cause chemical modifications altering their functional attributes and consequently, their ability to prevent droplet aggregation. Noncovalent interactions may also occur, for instance, hydrophobic interactions between volatile oxidation products and hydrophobic regions of proteins [54]. The higher concentration of volatile for all emulsions, using BSFL as emulsifier, might have contributed to the formation of residual lipid content in the extract. The high temperature and pre-treatment used for protein extraction could have accelerated the lipid oxidation and production of hexanal and n-heptanal, which were close to 0 for CAS emulsion. However, this is the first time that alkaline BSFL protein concentrate and BSFL subjected to OH and ultrasound treatment were evaluated for their ability to provide oxidative stability as emulsifiers. As previously stated, PV values for BSFL protein concentrate, after 10 days, were lower when compared with treated (OH and UOH) samples; however, further studies must be performed in order to fully comprehend how the different pre-treatments can cause lipid oxidation and lower the emulsion stability.

## 4. Conclusions

Emulsions stabilized with BSFL-OH showed the most promising physical stability, which was confirmed by TSI value and the hydrodynamic diameter. Modifications on the secondary structure could be noticed by FTIR after pre-treatment with OH alone and the combination of ultrasound and OH. This indicated that the treatment the influenced the techno-functional properties of proteins. The improved capacity of BSFL-OH to stabilize O–W emulsion was also confirmed by the lowest IFT reported in the pendant drop test. This is the first time that a BSFL sample was treated with OH in order to increase protein content and improve emulsifying properties. Therefore, further research is necessary to investigate how these treatments can work on the functionality of BSFL proteins. For the oxidative stability, BSFL, BSFL-OH and BSFL-UOH emulsions showed higher oxidation when compared to CAS. Peroxide value had an increasing pattern over time when compared to CAS reference emulsion, and the highest oxidation was reported for BSFL-UOH samples, which means that OH and ultrasound treatment in the specific method herein reported did not improve oxidative stability. As oxidation was already occurring for BSFL protein concentrate, antioxidants might be recommended during protein extraction. However, other parameters, such as using the same techniques as pre-treatment, can be applied in order to investigate how physical and oxidative stability can be improved. For further studies, the combination of BSFL protein and surfactants, such as phosphatidylcholine and lecithin, can improve the efficiency in protecting emulsified lipids against oxidation. In addition, the protein concentration and hydrolysis of BSFL protein might play an important role in lowering the interfacial tension and improve the interfacial stability of O–W emulsions.

## Data Availability

The data presented in this study are available on request from the corresponding author upon reasonable request.

## Supplementary Materials

The following are available online at https://www.mdpi.com/article/10.3390/foods10122977/s1, Figure S1: Content of secondary volatile in all emulsion during 10 days of storage. (A). 1-penten-3-one; (B) pentanal; (C) 1-penten-3-ol; (D) *(E)*-2-pentenal (E) *(E)*-2-hexenal (F) (Z)-4-heptenal (G) *(E)*-2-heptenal (H) 1-octen-3-ol (I) octanal (J) *(E,E)*-2,4-heptadienal (K) 2-octenal (L) *(E,E)*-2,4- decadienal.
