# Supplementary material for: Physical and Oxidative Stability of Low-Fat Fish Oil-in-Water Emulsions Stabilized with Black Soldier Fly (Hermetia illucens) Larvae Protein Concentrate
Source: Foods. 2021 Dec 3;10(12):2977. doi: 10.3390/foods10122977 (PMC8701752; doi:10.3390/foods10122977)
Supplement: Supplementary file 1 [file foods-10-02977-s001.zip › foods-1468030-supplementary.pdf]

Supplementary

Physical and Oxidative Stability of Low-Fat Fish Oil-in-Water Emulsions Stabilized with Black Soldier Fly (*Hermetia illucens*) Larvae Protein Concentrate

Lucas Sales Queiroz [1,2], Federico Casanova [1], Aberham Hailu Feyissa [1], Flemming Jessen [1], Fatemeh Ajalloueian [3], Italo Tuler Perrone [4], Antonio Fernandes de Carvalho [2,*], Mohammad Amin Mohammadifar [1], Charlotte Jacobsen [1] and Betül Yesiltas [1,*]

[1] National Food Institute, Technical University of Denmark, 2800 Kongens Lyngby, Denmark; lusaqu@food.dtu.dk (L.S.Q.);feca@food.dtu.dk (F.C.); abhfe@food.dtu.dk (A.H.F.); fjes@food.dtu.dk (F.J.); moamo@food.dtu.dk (M.A.M.); chja@food.dtu.dk (C.J.)
[2] Departamento de Tecnologia de Alimentos, Universidade Federal de Viçosa (UFV), 36570-900 city of Viçosa,, Brazil
[3] Center for Intelligent Drug Delivery and Sensing Using Microcontainers and Nanomechanics (IDUN), Department of Health Technology, Technical University of Denmark, 2800 Kongens Lyngby, Denmark; faaj@dtu.dk
[4] Departamento de Ciências Farmacêuticas, Universidade Federal de Juiz de Fora (UFJF), 36036-900 Rua José Lourenço Kelmer, São Pedro, Juiz de Fora, , Brazil; italotulerperrone@gmail.com
* Correspondence: antoniofernandes@ufv.br (A.F.d.C.); betye@food.dtu.dk (B.Y.)

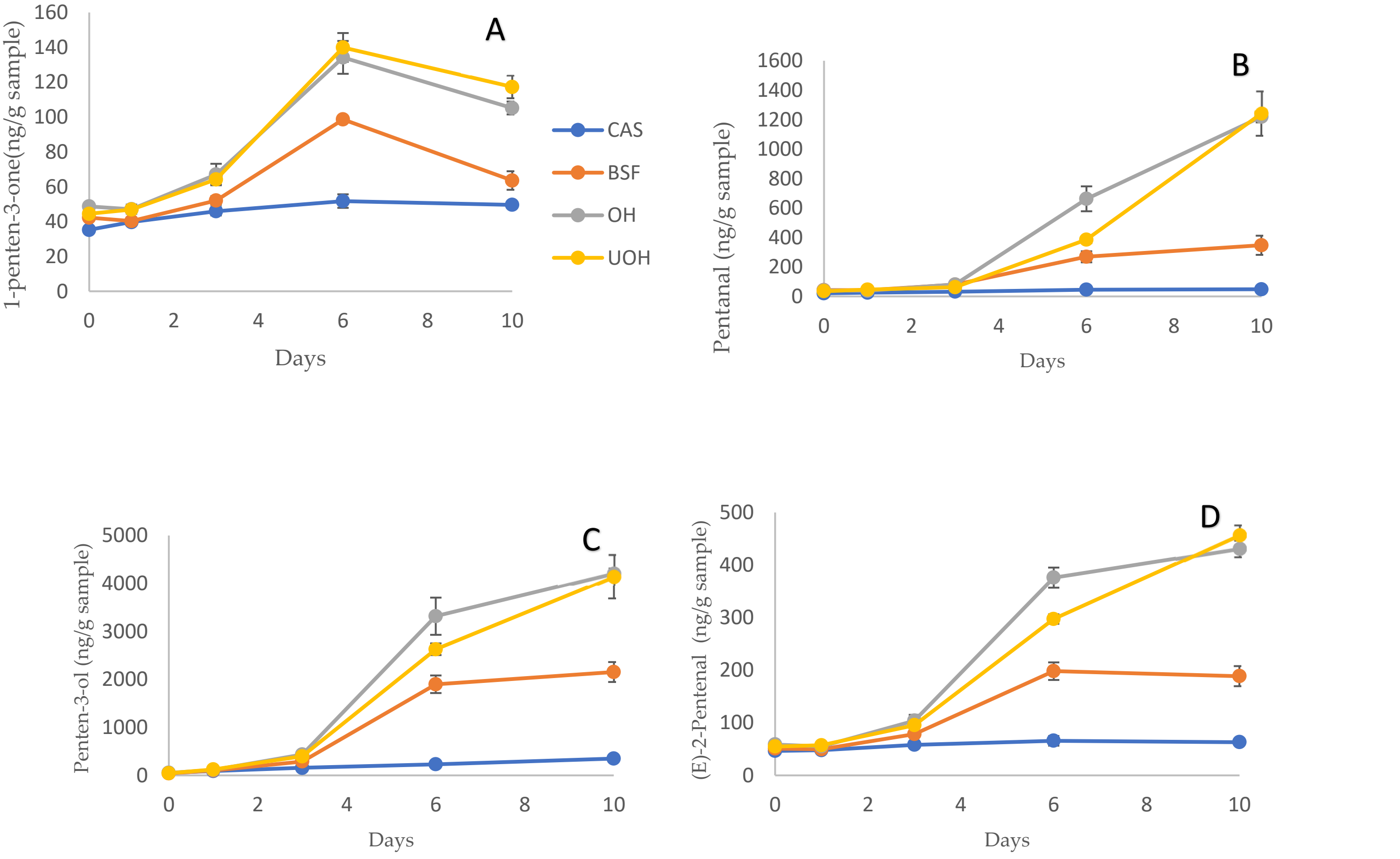

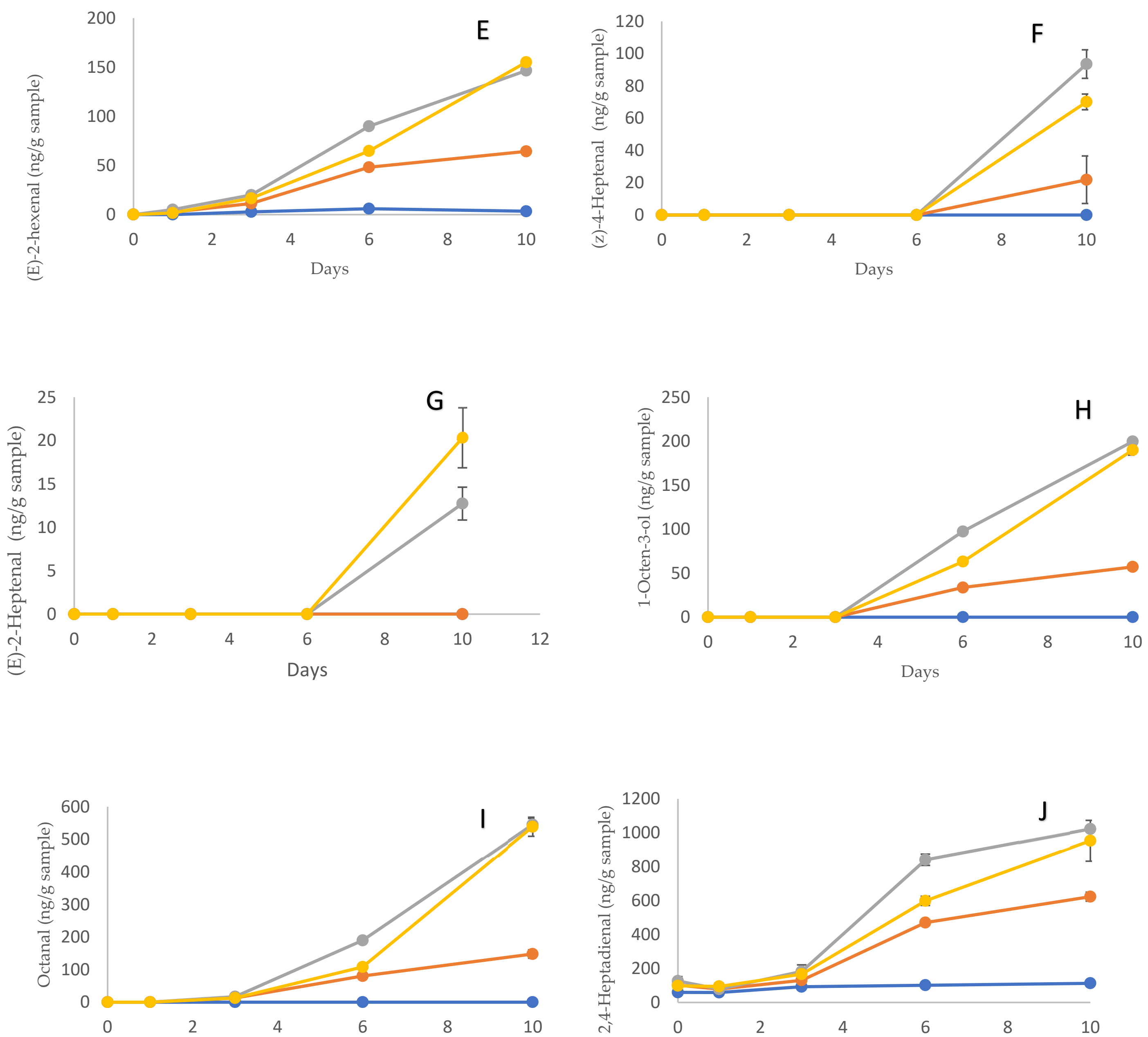
E
(E)-2-hexenal (ng/g sample)
0
50
100
150
200
0
2
4
6
8
10
Days
F
(z)-4-Heptenal (ng/g sample)
0
20
40
60
80
100
120
0
2
4
6
8
10
Days
G
(E)-2-Heptenal (ng/g sample)
0
5
10
15
20
25
0
2
4
6
8
10
12
Days
H
1-Octen-3-ol (ng/g sample)
0
50
100
150
200
250
0
2
4
6
8
10
Days
I
Octanal (ng/g sample)
0
100
200
300
400
500
600
0
2
4
6
8
10
Days
J
2,4-Heptadienal (ng/g sample)
0
200
400
600
800
1000
1200
0
2
4
6
8
10
Days

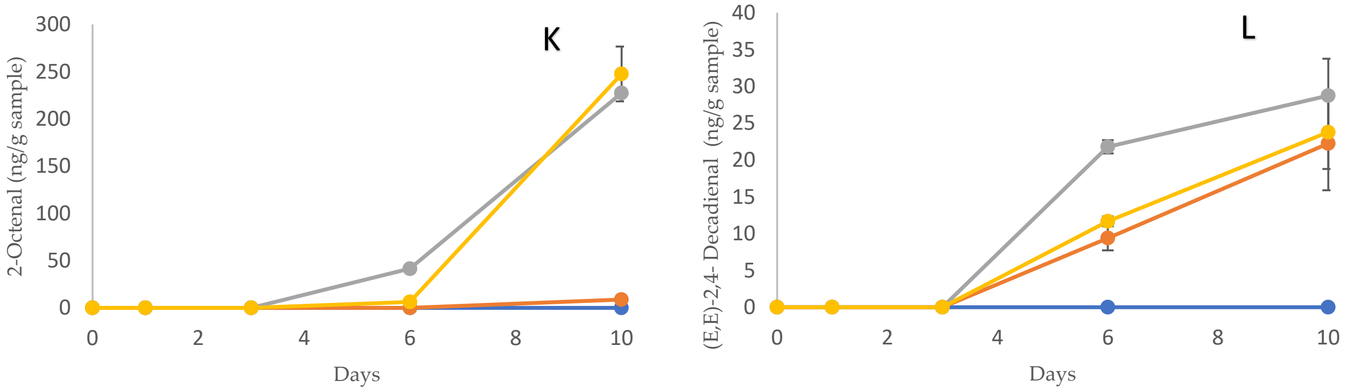

Figure S1. Content of secondary volatile compounds, in sodium caseinate (CAS), black soldier fly larvae (BSF), black soldier fly ohmic heating treatment, black soldier fly ultrasound and ohmic heating treatment (UOH) emulsion during 10 days of storage. (**A**) 1-penten-3-one; (**B**) Pentanal.; (**C**) Penten-3-ol.; (**D)** (E)-2-Pentenal **.(E)** (E)-2-hexenal **(F)** (z)-4-Heptenal **(G)** (E)-2-Heptenal **(H)** 1-Octen-3-ol **(I)** Octanal **(J)** 2,4-Heptadienal **(K)** 2-Octenal **(L)** (E,E)-2,4- Decadienal**.**
